# Could an optimally fitted categorization of difference between multi-disease score and multi-symptom score be a practical indicator aiding in improving the cost-effectiveness of healthcare delivery for older adults in developing countries?

**DOI:** 10.1186/s12939-023-02024-z

**Published:** 2023-10-11

**Authors:** Yuhong Wang, Guoying Guan, Ying xue, Jingyu Zhang, Zhe Cui, Hui Han

**Affiliations:** Department of Geriatrics, The First Affiliated Hospital of Harbin Medical University, No.23 You-Zheng Street, Harbin, China

**Keywords:** Age-related health decline, Aging-associated multimorbidity, Multi-disease, Multi-symptom, Metrics, Cost-efficiency of healthcare delivery

## Abstract

**Background:**

Physio-psycho-socioeconomical health comprehensively declines during aging, the complexity of which is challenging to measure. Among the complexity, multiple chronic disorders continuously cumulated during aging, further aggravating the challenge.

**Methods:**

A population-based survey on Comprehensive Ageing Health Assessment was conducted in older adults (age >  = 60) enrolled from hospital settings and community settings in 13 working centers in six subnational regions in China. Cross-sectional datasets of 8,093 older participants with approximately complete assessment results were collected for the present analysis. Individual’s multi-disease or multi-symptom was respectively scored by summing coexistent multiple diseases or multiple symptoms by respective weighting efficient for Self-Rated Health (SRH). Individual’s age-dependent health decline was further summed of four SRH-weighted scores for daily function (activity of daily life, ADL), physical mobility (an average of three metrics), cognitive function (mini mental state examination, MMSE) and mental being (geriatric depression scale, GDS) plus multi-disease score (MDS) and multi-symptom score (MSS).Multi-disease patten among 18 diseases or multi-symptom pattern among 15 symptoms was latent-clustered in the older adults, the optimal outcome of which was categorized into high, moderate or low aging-associated clusters, respectively. Percentage distribution was compared between overall health decline score and multi-disease pattern cluster or multi-symptom patten cluster. A new variable of difference between MDS and MSS (hereinafter terming DMM) that displayed linear variation with socioeconomic factors was further fitted using multilevel regression analyses by substantial adjustments on individual confounders (level-1) and subnational region variation (level-2).

**Results:**

Consistent gradient distribution was shown between health decline and multimorbidity pattern cluster in the older adults. DMM was found linearly varied with personal education attainment and regional socioeconomic status. Using optimally fitted stratification of DMM (DMM interval = 0.02), an independent U-shaped interrelated tendency was shown between health decline, multi-disease and multi-symptom, which could be well explained by regional disparities in socioeconomic status.

**Conclusion:**

Newly developed metrics for age-dependent health decline and aging-associated multimorbidity patten were preliminarily validated from within. The new variable of optimally fitted categorization of DMM might function as a practical indicator aiding in improving the cost-effectiveness and reduce inequity of healthcare delivery for older adults in developing countries.

**Supplementary Information:**

The online version contains supplementary material available at 10.1186/s12939-023-02024-z.

## Introduction

The age-dependent multidimensional health decline is challenging to assess due to its internal complexity and contextual cumulation [1–3]. Ageing per se contextually declines multidimensional health in an old adult by incorporating deteriorated physio-pathological function, disordered psychological being and lower socioeconomic status [4, 5]. Moreover, human life expectancy is ongoing increased due to continued advance of industrialization-based health care and disease prevention, leading to remarkedly global aging population. The lifelong healthcare not only retarded the overall age-dependent health decline, but cumulated chronic conditions including both diseases and symptoms [6, 7], generally termed as multimorbidity, with extremely heterogeneous patterns [2, 7–9]. Such heterogeneously coexistence of multiple chronic conditions does not indicate the rapid onset of death or markedly decreased function [10, 11] but substantially enhance the complexity of multidimensional health decline during aging. Therefore, we had supposed that multimorbidity as the most popular phenomenon of aging during the industrialization era which had addressed expanded lifespan might imply some uncovered evidence to reflect the industrialization-based contemporary healthcare and individual-based accumulative multimorbidity in old adults, However, complicated being of multimorbidity was, it had not been well defined and/or qualified worldwide. It was documented that complicated background during contextual aging could be better explained by using cumulative or integrative measures [12]. Latent cluster analysis was used to help to disentangle the complexity of multidimensional health status during aging [13]. To our best knowledge, no modelling was fitted on the multidimensional complexity of age-dependent health decline from within. Using national datasets from Comprehensive Ageing Health Assessment (CAHA) in China, we conducted a stepwise data analysis by the following four major steps: (1) We measured overall health decline in the older adults by summing six SRH-adjusted scores on five domains, and profiled it nationally and sub-nationally. (2) We clustered multimorbidity (multi-disease and multi-symptom) pattern using latent-based analysis, and categorized them into three aging-associated subgroups. (3) For the newly developed metrics, we validated them from within by gradient distribution comparison as no similar measurements existed for validation. (4) We further analyzed these aging-dependent multimorbidity-related innovative measurements with national/subnational socioeconomical variables to seek some intrinsic correlation between bio-physio-patho-psychological dimension of health and socioeconomical dimension of health within the complexity of age-dependent health decline.

## Methods

### A national survey on Comprehensive Ageing Health Assessment (CAHA) in China

A national face-to-face survey of Comprehensive Assessment of Ageing Health was launched in China in October 2011 using a goal-oriented scale that combined dozens of widely used scales. The compound scale comprised eight components, including demographics, physio-pathological status (coexistence of claimed multi-disease and multi-symptom), daily life function, lifelong lifestyles, cognitive status, mental being, socioeconomic status and healthcare utilization based on the framework of Comprehensive Geriatric Assessment (CGA) (The English version of the Scale is shown in Supplementary File S1). Datasets of eligible 8,093 Chinese older adults aged over 60 harboring approximate complete surveyed results were used for the present analysis. The protocol of the national sampling from both hospitals and communities in six subnational regions is shown in Supplementary File S2. All face-to-face interviewers were professional geriatricians and gerontologists from respective study centers. Written approval was required from all respondents.

### Scoring multimorbidity individually

The existence of 18 claimed chronic diseases and 15 claimed chronic symptoms were collected from the datasets. Each disorder (disease/symptom) was weighted by Self-Rated Health (SRH) using a dichotomized outcome (positive/negative) in the present population-based older samples [14]. The odds ratio (OR) with the β coefficient was estimated by comparing positive self-rated health vs. negative self-rated health with or without a particular disorder [15] to acquire a weighting coefficient for each condition. A statistically significant β coefficient was defined to weight the extent to which a coexistent disorder impacted overall health. Using these weighting coefficients, individual’s coexistence of multi-diseases or multi-symptoms was respectively summed, termed as multi-disease score (MDS) or multi-symptom score (MSS). Heterogeneous patten of multimorbidity (multi-disease and multi-symptom) was globally measured as a latent (here was a cluster) by using latent cluster analysis (LCA) [16] in the older adults. As low prevalence of 18 chronic diseases or 15 symptoms would produce hundreds of multimorbidity combination patterns, clusters harboring disorders with < 5% prevalence were removed to ensure successful LCA fitting [17]. Pre-setting 2 ~ 10 class as fitting range, the present LCA produced a bundle of clusters, which was further optimized based on the smallest Bayesian information criterion (BIC). The optimal cluster for multi-disease or multi-symptom was respectively described by coexistent disease/symptom prevalence variation compared to its pooled prevalence [18].

### Scoring age-related health decline

Age-dependent health decline was scored by summing a function decline scored by activities of daily living (ADL), a physical mobility decline scored by an average time for three mobility tests (stand, balance and walking speed), a cognitive decline measured by the Mini-Mental State Examine (MMSE) and a likelihood of depression using the geriatric depression scale (GDS), and a coexistence of multimorbidity by scored multi-disease adding scored multi-symptom. Except for the MDS and MSS, the remaining scores could be directly obtained from the national dataset. For an old adult, each domain decline was weighted by his/her SRH prior to summation. Also, normalization was employed prior to summing to avoid bias [18, 19].

### Profiles of age-dependent health decline in the older adults nationally and sub-nationally

The national and subnational health decline score was profiled by age, gender, setting, residency, educational attainment and personal income, two latter variables of which are regarded as conventional proxies of socioeconomic status during ageing [20, 21]. The population-based older adults were stratified by ageing-related health decline scores to create three subgroups: a subgroup harboring low health decline, a subgroup harboring moderate health decline, and a subgroup harboring high health decline.

### Profiles of multi-disease score and multi-symptom score in the older adults nationally and sub-nationally

The national and subnational multi-disease (using MDS) and multi-symptom (using MSS) profiles were profiled by age, gender, setting, residency, educational attainment and personal income. Data mining was employed to reveal a novel variable of the difference between MDS and MSS, herein termed DMM (MDS—MSS). The distributed correlation was profiled between DMM and each of three socioeconomic variables individually and regionally.

### Profiles of aging-associated clusters for multi-disease pattern or multi-symptom pattern in the older adults nationally

The national multi-disease pattern clusters and multi-symptom clusters were categorized into three aging-associated subgroups, i.e., low aging-associated cluster, moderate aging-associated cluster and high aging-associated cluster, for multi-disease patten or multi-symptom pattern respectively.

### Gradient distribution comparison between age-dependent health decline score and aging-associated cluster for multimorbidity pattern

The percentage distribution was compared between the subpopulations categorized by age-dependent health decline score and the subpopulations categorized by aging-associated cluster for multi-disease pattern or for multi-symptom pattern.

### Optimize a newly mined variable to disentangle the complexity between health decline score, multi-disease score and multi-symptom score in the older adults using multilevel regression

Using datamining, a newly variable of difference between MDS and MMS arose, which displayed a socioeconomic interrelationship. Using two-level regression model, associated multi-disease score as well as multi-symptom score was “disentangled” by substantially adjusting low-level individual confounders (age, sex, setting, residency, educational attainment, income) and high-level regional variation. The DMM was iteratively fitted from its lowest value of -0.04, to its highest value of + 0.04 at different intervals. The optimal fit was considered when the independent interrelationship between age-related health decline and multi-disease score as well as multi-symptom score could be well explained.

### Statistical analysis

**Fig. 1:**
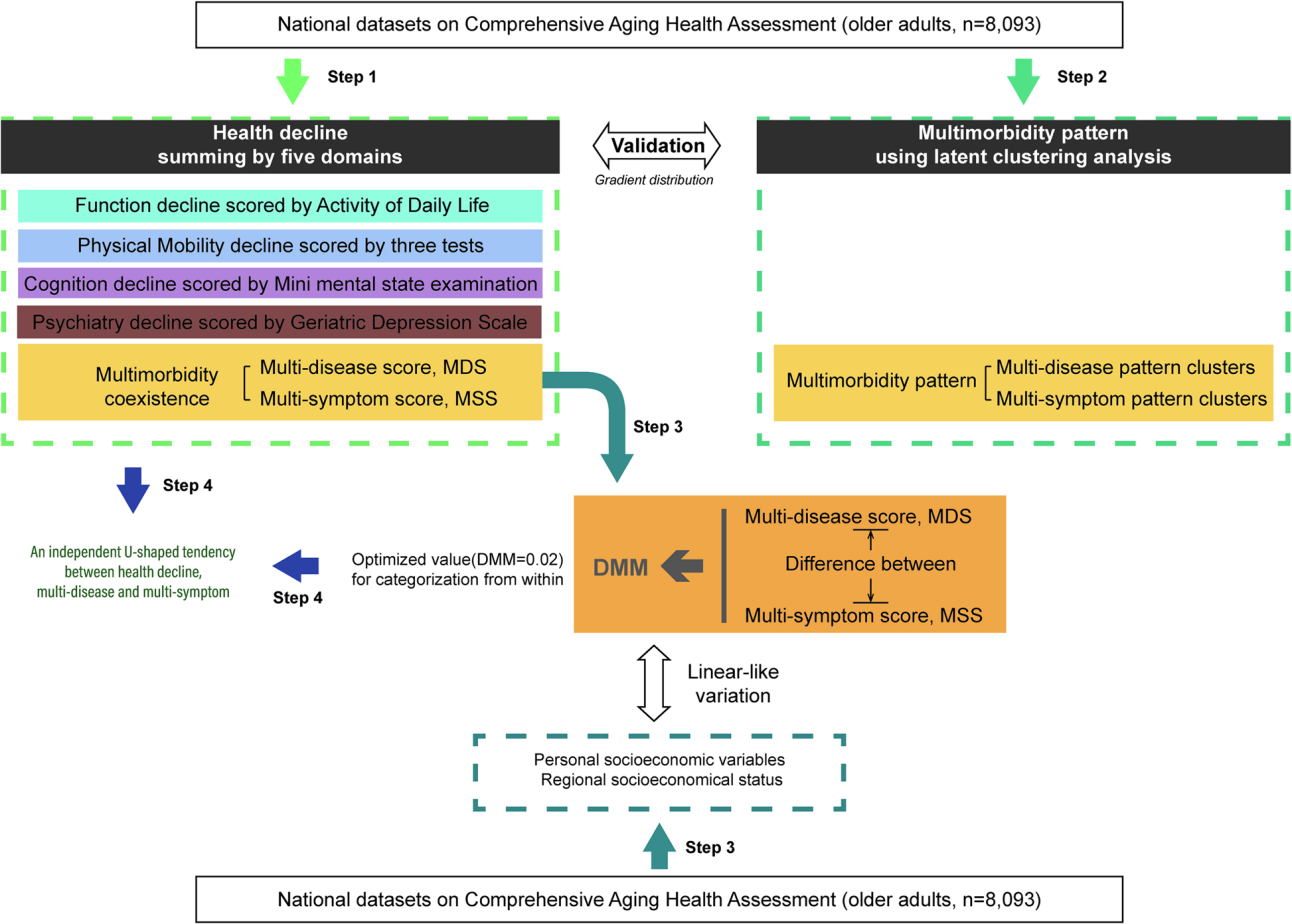
The flow diagram of the stepwise data analysis in the older adults in China. (To better display the outcome-dependent stepwise data analysis on the present national datasets, findings for each step was shown in enclosed bracket respectively in Results)

Data mining was conducted step by step according to the respective references. The flow diagram for the stepwise roadmap is shown in Fig. 1. All statistical analyses were performed using SAS (Version 9.4; SAS Institute, Cary, North Carolina, USA) and R (3.5.3).

## Results

### Sociodemographic characteristics of the older Chinese adults used for data mining (Upper/Lower rectangle shown in Fig. 1)

The present data analysis used datasets from 8,093 older participants with high-quality data. The majority (77.41%) were registered as urban residents based on the Chinese Hu-Kou system, indicating the rapid urbanization in China. In addition, as the National Project was carried out in respective metropolitan centers, only a small proportion of rural elderly individuals could be successfully enrolled from remote rural communities, which unavoidably led to sampling bias. Consistently, the present representative aging population was characterized by a high proportion of people who had a high education level (educational attainment more than 9 years, > 50%). Notably, we included only those within the 90% quartile of claimed income in the current datasets, as the claimed income varied substantially, with few reporting incomes beyond “reasonable range”. Therefore, the sample size is slightly smaller for the income quartile. The sociodemographic characteristics of the elderly population in China are outlined in Table 1.

**Table 1:** Sociodemographic characteristics of the older adults in China, by region (% of the indicated population)

Category	Total (Nation)	North # of samples	Northeast	East	Middle	Southwest	Northwest
Age (years ± SD)	71.97 ± 7.82	71.83 ± 7.84	75.23 ± 7.17	69.53 ± 6.75	70.76 ± 7.44	73.11 ± 7.99	70.87 ± 7.76
	Percentage population (%)						
Age							
60–69	41.63	41.77	21.79	55.04	46.32	37.76	48.69
70–79	39.35	40.11	48.29	35.43	38.95	37.37	37.93
≥ 80	19.02	18.12	29.92	9.53	14.73	24.87	13.39
Sex							
Female	46.72	44.35	42.93	47.48	48.99	50.76	47.11
Male	53.28	55.65	57.07	52.52	51.01	49.24	52.89
Residency (by Kuhou)							
Urban	77.41	90.3	96.59	52.25	64.15	64.93	75.56
Rural	22.59	9.7	3.41	47.75	35.85	35.07	24.44
Sample Source							
Community	48.23	45.06	49.76	51.08	28.85	66.04	44.39
Hospital	51.77	54.94	50.24	48.92	71.15	33.96	55.61
Years of Education							
None	18.47	17.8	6.34	22.7	21.11	22.15	15.28
Low(< 5y)	20.17	19.03	7.15	33.15	26.54	19.38	18.31
Moderate(5-9y)	8.56	5.27	0.98	5.77	4.52	22.15	3.82
High (> 9y)	52.79	57.9	85.53	38.38	47.83	36.32	62.58
Individual Income (by Quartile)							
Lowest	24.11	16.96	2.28	29.07	22.6	43.11	24.45
Second	36.85	31.76	30.24	40.04	50.14	32.18	44.78
Third	15.87	19.65	25.04	9.51	18.02	7.98	14.56
Highest	23.17	31.63	42.44	21.39	9.24	16.73	16.21

### Scoring personal multi-disease and multi-symptom coexistence by adjusting SRH (Step 1 shown in Fig. 1, yellow block)

**Fig. 2:**
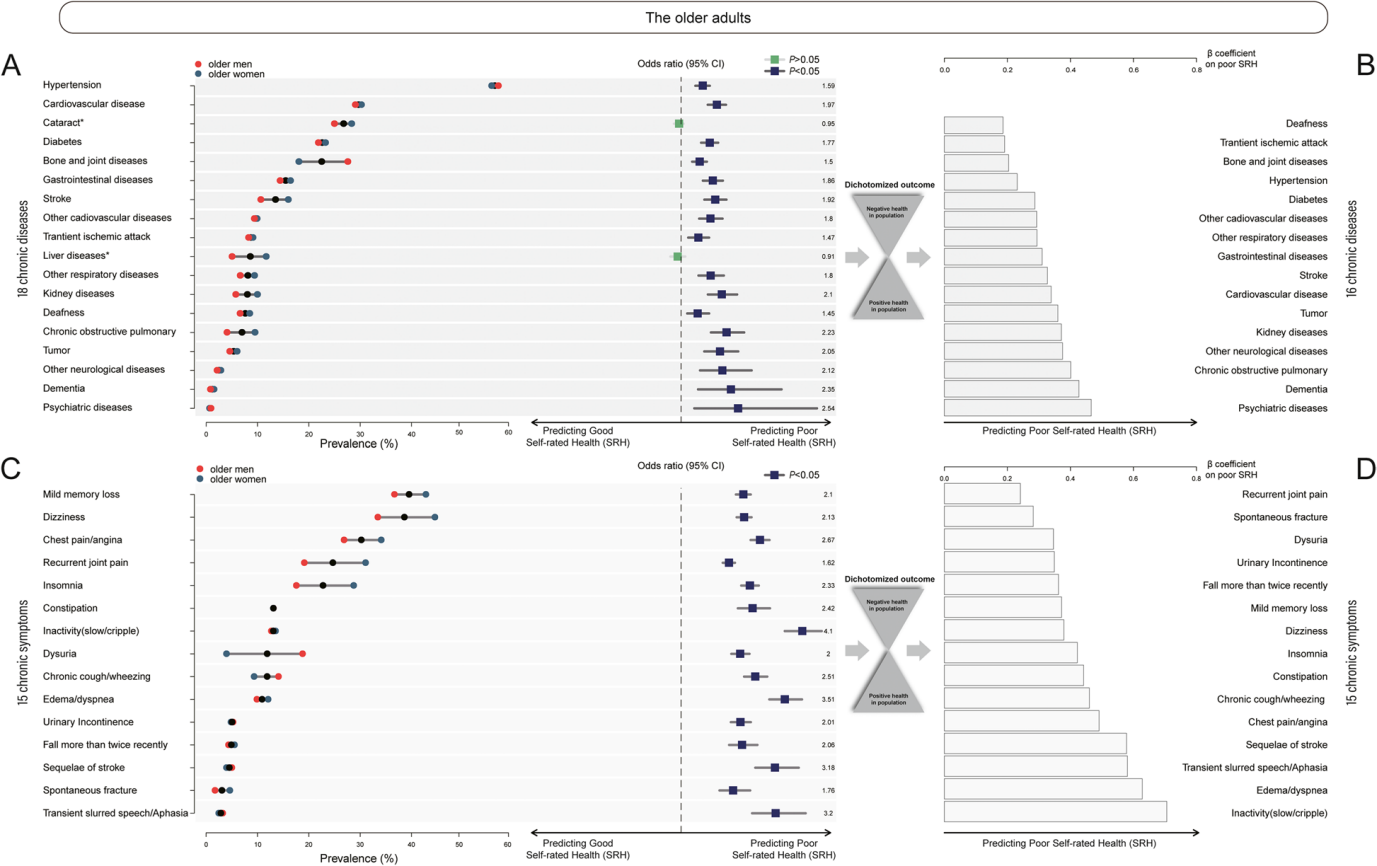
Sex-dependent prevalence of 18 chronic diseases and 15 chronic symptoms with respective impact on Self-Rated Health (SRH). **A** Sex-dependent prevalence of 18 chronic diseases with respective Odds Ratio on negative SRH. **B** β coefficient of 16 chronic diseases that significantly led to negative SRH by dichotomizing population claiming either positive SRH or negative SHR. **C** Sex-dependent prevalence of 15 chronic symptoms with respective Odds Ratio on negative SRH. **D** β coefficient of 15 symptoms that significantly led to the negative SRH by dichotomizing population claiming either positive SRH or negative health. *Asterisks indicate the two diseases were of insignificant impact on negative SRH (*P* > 0.05)

The sex-dependent prevalence of 33 age-related conditions (18 common chronic diseases and 15 common chronic symptoms) was initially profiled with the respective Odds Ratio (OR) weighted by SRH, and the overall findings were consistent with previous studies [22]. A notable profiling difference was observed between the prevalence of a specific disorder and the significant negative impact on SRH of the disorder, indicating that the most prevalent disorder was far from the most detrimental factor with respect to SRH (Fig. 2). The SRH-related β coefficient of a single disease or symptom was used to weight the multi-disease or multi-symptom coexistence in the present older Chinese adults. Using this weighting system, personal multi-disease and multi-symptom was scored by the sum of the weights of an individual’s coexisting diseases or symptoms respectively.

### Profiling of the multi-disease score (MDS) and the multi-symptom Score (MSS) nationally and sub-nationally (Step 2 shown in Fig. 1, yellow block)

Using the obtained MDS and MSS, national and sub-national multi-disease and multi-symptom status were profiled by age, gender, setting, residency, educational attainment and personal income (Table 2). An age-related increase was shown in the MDS and MSS across all six subnational regions. Moreover, the largest increment appeared in the old-old subgroup aged over 80. Globally, older Chinese men report a slightly higher number of diseases than older women, and elderly Chinese women report a notably higher number of symptoms than older men, which supports the conventional male-female health-survival paradox [23]. Multi-disease and multi-symptom scores were similar between urban and rural residents. Moreover, both were remarkably elevated in hospital settings compared to community settings, strongly reflecting the “real world” situation. Personal income displayed little correlation with multi-disease score or multi-symptom score. Interestingly, unlike in most developed countries, where multi-disease morbidity increases with decreased educational attainment [20], our findings showed that in the assessed old adults, multi-disease score increased with increased educational attainment across the nation, with slight variation. Notably, such a phenomenon has also been seen in other developing countries, such as India [24] and Brazil [25], both of which harbour remarkable socioeconomic inequities [26]. This indicates a reversal of the commonly observed correlation between increased multi-disease burden and low educational attainment in older adults in industrialized countries [20]. However, we did find a counter-correlation between the increased multi-symptom score and lower educational attainment in most of the same population, indicating an interesting “paradoxical” trajectory between multi-disease score and multi-symptom score in relation to personal education. Of note, the national face-to-face assessment requires professional diagnosis for all claimed multiple diseases as a reference. This requirement ensures that the claimed diseases represent for the extent of disease awareness with a high likelihood of management based on available healthcare. In contrast, all reported symptoms were based on self-report. Therefore, we sought to calculate the difference between the scored multi-disease and multi-symptom, terming DMM, as a new variable that might be with potential socioeconomic interrelationship.

**Table 2:** Characteristics of multi-disease score (MDS) and multi-symptom score (MSS) in the older population, by subnational regions

Variable	National		North		Northeast		East		Middle		Southwest		Northwest	
	MDS	MSS	MDS	MSS	MDS	MSS	MDS	MSS	MDS	MSS	MDS	MSS	MDS	MSS
Age (years)														
60–69	0.46 ± 0.38	0.76 ± 0.76	0.48 ± 0.40	0.61 ± 0.68	0.57 ± 0.42	0.86 ± 0.97	0.43 ± 0.36	0.61 ± 0.58	0.47 ± 0.31	0.67 ± 0.58	0.42 ± 0.40	1.10 ± 0.94	0.39 ± 0.35	0.85 ± 0.74
70–79	0.62 ± 0.46	0.99 ± 0.90	0.69 ± 0.48	0.86 ± 0.85	0.60 ± 0.43	1.04 ± 0.97	0.47 ± 0.41	0.56 ± 0.69	0.59 ± 0.37	0.87 ± 0.73	0.57 ± 0.49	1.39 ± 1.00	0.550.44 ±	1.04 ± 0.83
≥ 80	0.85 ± 0.56	1.51 ± 1.13	1.00 ± 0.56	1.45 ± 1.13	0.80 ± 0.54	1.73 ± 1.21	0.60 ± 0.50	0.99 ± 0.83	0.64 ± 0.41	1.11 ± 0.93	0.82 ± 0.54	1.79 ± 1.12	0.67 ± 0.61	1.14 ± 1.02
Sex														
Female	0.56 ± 0.45	1.07 ± 0.94	0.62 ± 0.47	0.98 ± 0.93	0.70 ± 0.45	1.36 ± 1.10	0.42 ± 0.39	0.67 ± 0.65	0.52 ± 0.34	0.80 ± 0.67	0.54 ± 0.47	1.42 ± 1.00	0.47 ± 0.43	1.04 ± 0.81
Male	0.63 ± 0.50	0.94 ± 0.94	0.70 ± 0.53	0.80 ± 0.86	0.63 ± 0.49	1.11 ± 1.10	0.50 ± 0.41	0.60 ± 0.67	0.57 ± 0.37	0.85 ± 0.77	0.63 ± 0.52	1.38 ± 1.09	0.50 ± 0.45	0.90 ± 0.84
Residence (Hukou)														
Urban	0.64 ± 0.49	0.99 ± 0.95	0.69 ± 0.51	0.88 ± 0.91	0.66 ± 0.48	1.21 ± 1.11	0.59 ± 0.41	0.71 ± 0.71	0.53 ± 0.38	0.80 ± 0.76	0.67 ± 0.52	1.36 ± 1.05	0.50 ± 0.47	0.96 ± 0.85
Rural	0.45 ± 0.38	1.04 ± 0.91	0.40 ± 0.40	0.83 ± 0.83	0.74 ± 0.33	1.54 ± 0.93	0.33 ± 0.33	0.54 ± 0.60	0.57 ± 0.33	0.87 ± 0.66	0.43 ± 0.41	1.47 ± 1.04	0.47 ± 0.35	0.98 ± 0.75
Setting														
Community	0.48 ± 0.43	0.94 ± 0.89	0.58 ± 0.47	0.85 ± 0.83	0.45 ± 0.37	0.69 ± 0.71	0.36 ± 0.37	0.58 ± 0.65	0.44 ± 0.35	0.67 ± 0.73	0.44 ± 0.41	1.27 ± 1.00	0.39 ± 0.40	0.87 ± 0.78
Hospital	0.71 ± 0.49	1.07 ± 0.99	0.74 ± 0.52	0.89 ± 0.95	0.87 ± 0.47	1.75 ± 1.18	0.57 ± 0.40	0.68 ± 0.67	0.58 ± 0.36	0.88 ± 0.72	0.86 ± 0.53	1.63 ± 1.08	0.57 ± 0.46	1.04 ± 0.85
Educational attainment (Stratification by years of schooling)														
Low (< 5y)	0.54 ± 0.45	1.17 ± 1.00	0.64 ± 0.49	1.01 ± 1.03	0.66 ± 0.35	1.53 ± 1.02	0.42 ± 0.40	0.73 ± 0.76	0.56 ± 0.33	0.96 ± 0.76	0.47 ± 0.44	1.45 ± 1.03	0.54 ± 0.44	1.20 ± 0.85
Moderate (5-9y)	0.57 ± 0.45	0.93 ± 0.89	0.60 ± 0.48	0.86 ± 0.89	0.60 ± 0.40	1.04 ± 0.88	0.43 ± 0.39	0.56 ± 0.62	0.55 ± 0.33	0.81 ± 0.67	0.62 ± 0.51	1.30 ± 1.07	0.50 ± 0.43	0.98 ± 0.81
High (> 9y)	0.64 ± 0.50	0.95 ± 0.92	0.70 ± 0.52	0.84 ± 0.85	0.66 ± 0.49	1.20 ± 1.13	0.52 ± 0.40	0.61 ± 0.59	0.53 ± 0.39	0.76 ± 0.73	0.70 ± 0.53	1.37 ± 1.04	0.47 ± 0.44	0.88 ± 0.79
Personal Income (Stratification by quartiles)														
Lowest	0.45 ± 0.39	1.07 ± 0.92	0.44 ± 0.40	0.86 ± 0.83	0.95 ± 0.31	1.71 ± 1.16	0.32 ± 0.32	0.56 ± 0.57	0.54 ± 0.34	0.89 ± 0.68	0.46 ± 0.40	1.38 ± 1.02	0.43 ± 0.35	0.97 ± 0.77
Second	0.59 ± 0.46	0.98 ± 0.93	0.67 ± 0.49	0.97 ± 1.03	0.65 ± 0.48	1.25 ± 1.10	0.54 ± 0.46	0.84 ± 0.82	0.56 ± 0.34	0.83 ± 0.77	0.55 ± 0.50	1.11 ± 0.94	0.48 ± 0.41	1.00 ± 0.82
Third	0.55 ± 0.43	0.90 ± 0.88	0.56 ± 0.45	0.80 ± 0.89	0.61 ± 0.45	1.05 ± 0.97	0.51 ± 0.37	0.52 ± 0.50	0.54 ± 0.38	0.81 ± 0.72	0.65 ± 0.48	1.35 ± 1.04	0.42 ± 0.41	0.83 ± 0.78
Highest	0.67 ± 0.51	1.07 ± 1.05	0.61 ± 0.47	0.88 ± 0.93	0.68 ± 0.49	1.30 ± 1.19	0.50 ± 0.38	0.53 ± 0.57	0.50 ± 0.38	0.73 ± 0.71	0.95 ± 0.57	1.73 ± 1.18	0.66 ± 0.56	1.04 ± 0.95

### Multi-disease pattern cluster and multi-symptom patten cluster nationally (Step 2 in shown Fig. 1, black block)

**Fig. 3:**
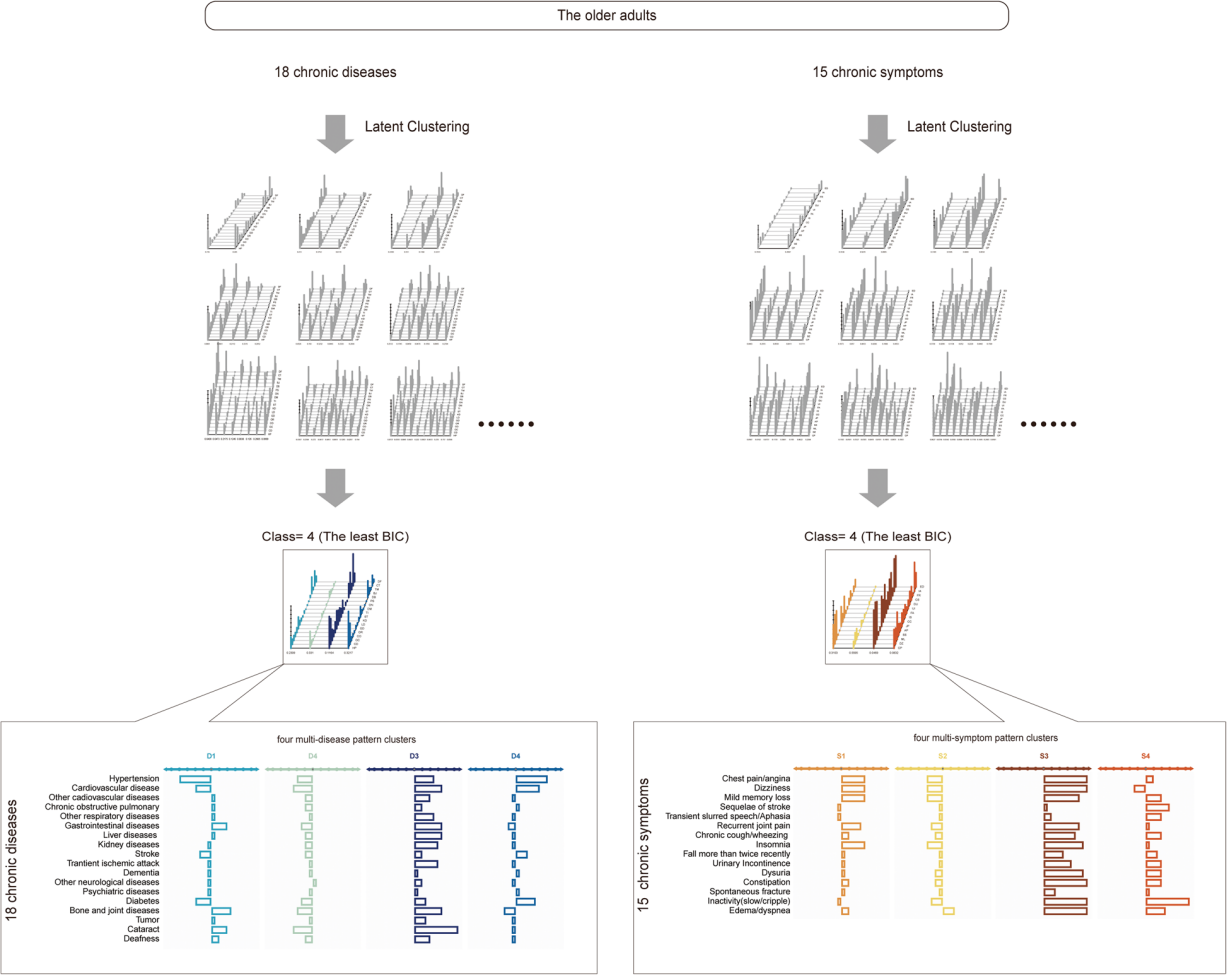
Heterogenous multi-disease patten and multi-symptom patten was latent-based categorized into different aging-associated clusters in the older adults using Latent clustering analysis (LCA). Multi-disease pattern and multi-disease patten was clustered from 2 to 10 classes using the Latent Cluster Analysis (LCA, grey pools) with optimization by lowest BIC. The four-class patten clustering was selected with the lowest BIC for both multi-disease patten and multi-symptom patten. Four outcome clusters of multi-disease patten **A** or multi-symptom patten **B** were further amplified, respectively. Each patten cluster was globally characterized by prevalence variation of each coexistent disorder compared to the average prevalence in the older Chinese adults evaluated in this study. Four multi-disease patten clusters were described as heavy aging-associated patten (D3), moderate-to-mild aging-associated patten (D4 and D1) or slight aging-associated patten (D2). Four multi-symptom patten were described as heavy aging-associated patten (S3), moderate-to-mild aging-associated patten (S4 and S1) or slight aging-associated patten (S2). Color palettes from light to dark indicate the increase of aging-associated aspect

To validate the present developed metrics on age-related health decline, multimorbidity was re-assessed by highly heterogeneous multimorbidity patterns in the same older samples via latent-based analysis. BIC-optimized clustering yielded four multi-disease pattern and four multi-symptom pattern as respectively optimal cluster for chronic coexistent pattern harboring the highest probability of coexistence in the present old population (The stepwise protocol of multi-disease/multi-symptom coexistent pattern estimation was shown in Fig. 3). As multimorbidity had been well characterized as an ageing-associated aspect [14, 16, 27–31], the four multi-disease pattern cluster were further categorized into high aging-associated multi-diseases patten cluster (D3), two moderate-to-mild aging-associated multi-disease patten clusters (D1 and D4) and one slight aging-associated multi-disease patten cluster (D2) displayed with a blue palette. Similarly, the four multi-symptom patten clusters were depicted as one high ageing-associated multi-symptom patten cluster (S3), two moderate-to-mild aging-associated multi-symptom patten clusters (S1 and S4) and one slight aging-associated multi-symptom patten cluster (S2), visualized with a brown palette.

### Summing health decline by adjusting SRH nationally and sub-Nationally (Step 1 shown in Fig. 1, black block)

Age-dependent health decline was summed by six SRH-adjusted scores covering five domains. The health decline score was profiled nationally and sub-nationally (Table 3). The lowest health decline was in the most affluent region (East). This outcome can be attributed largely to the favorable coastal climate, high education levels and advanced healthcare services in East China. Of note, Northeast China, which has a relative low socioeconomic level, displayed remarkably low health decline. The setting for the community sampling in this subnational region was randomly located at a top university, resulting in the recruitment of a group of retired professors, which may have skewed the results to produce this “unmatched” phenomenon. The highest health decline was seen in Southwest China, likely due to this region having both the highest proportion of the oldest adults and the lowest regional socioeconomic status. Regarding the two socioeconomic confounders (educational attainment and personal income), the lowest education level was seen in the subgroup with the largest health decline; as an exception, in the most affluent region, East China, the health decline disparity was very narrow.

**Table 3:** Profiles of age-dependent health decline (score) in the population-based older adults, by subnational region

Subnational Region (GDP *per* capita/Thousand Yuan, in 2012)	National (38.901)	North (80.495)	Northeast (32.817)	East (81.788)	Middle (34.096)	Southwest (33.428)	Northwest (26.120)
Age-dependent health decline							
Overall score	0.18 ± 2.31	0.21 ± 2.40	-0.58 ± 1.94	-0.62 ± 2.21	0.19 ± 2.03	0.88 ± 2.39	-0.42 ± 1.86
Age(years)							
60–69	-0.75 ± 1.82	-0.97 ± 1.65	-1.11 ± 1.88	-0.88 ± 2.15	-0.61 ± 1.75	-0.21 ± 1.94	-0.86 ± 1.74
70–79	0.16 ± 2.09	0.25 ± 2.12	-0.96 ± 1.61	-0.79 ± 2.02	0.46 ± 1.98	0.76 ± 2.08	-0.35 ± 1.82
≥ 80	1.67 ± 2.81	2.20 ± 2.91	0.25 ± 2.18	0.76 ± 2.80	0.91 ± 2.45	2.27 ± 2.71	0.09 ± 2.23
Sex							
Female	0.11 ± 2.30	0.14 ± 2.49	-0.42 ± 2.09	-0.81 ± 2.16	-0.12 ± 1.93	0.82 ± 2.21	-0.39 ± 1.85
Male	0.04 ± 2.34	0.05 ± 2.33	-0.75 ± 1.82	-0.57 ± 2.29	0.19 ± 2.15	0.74 ± 2.60	-0.67 ± 1.88
Residency (Hukou)							
City	0.06 ± 2.34	0.15 ± 2.43	-0.62 ± 1.94	0.23 ± 2.42	-0.28 ± 2.03	0.63 ± 2.48	-0.57 ± 1.87
Rural	0.12 ± 2.24	-0.47 ± 2.09	-0.31 ± 2.05	-1.65 ± 1.49	0.60 ± 1.96	1.08 ± 2.25	-0.44 ± 1.90
Source (setting)							
Community	-0.41 ± 1.97	-0.25 ± 1.97	-1.42 ± 1.20	-1.74 ± 1.37	-1.03 ± 1.65	0.27 ± 2.08	-0.97 ± 1.59
Hospital	0.52 ± 2.52	0.36 ± 2.67	0.20 ± 2.20	0.39 ± 2.41	0.46 ± 2.04	1.78 ± 2.68	-0.20 ± 2.00
Educational Attainment (Years)							
Low(< 5y)	0.69 ± 2.43	0.78 ± 2.86	0.04 ± 1.93	-0.75 ± 2.27	0.79 ± 1.88	0.99 ± 2.15	0.19 ± 2.06
Moderate(5-9y)	-0.08 ± 2.30	-0.12 ± 2.32	-0.79 ± 1.40	-0.93 ± 2.27	0.17 ± 2.01	0.52 ± 2.59	-0.54 ± 1.80
High (> 9y)	-0.15 ± 2.22	-0.02 ± 2.21	-0.65 ± 1.98	-0.46 ± 2.09	-0.45 ± 2.02	0.68 ± 2.58	-0.78 ± 1.77
Personal Income (RMB Yuan)							
Lowest quantile (< 1800)	0.13 ± 2.16	-0.21 ± 2.29	0.82 ± 2.09	-1.46 ± 1.66	0.72 ± 1.97	0.59 ± 2.06	-0.37 ± 2.00
Second low quantile (1800–3000)	-0.03 ± 2.38	0.31 ± 2.82	-0.44 ± 2.06	-0.38 ± 2.50	-0.05 ± 1.94	0.05 ± 2.36	-0.51 ± 1.65
Third low quantile (3001–4000)	-0.46 ± 2.09	-0.58 ± 2.14	-0.93 ± 1.66	-0.31 ± 2.32	-0.28 ± 1.99	0.52 ± 2.34	-1.02 ± 1.69
Highest quantile (> 4000)	-0.01 ± 2.62	-0.40 ± 2.62	-0.50 ± 2.05	-0.28 ± 2.21	-0.29 ± 2.16	1.91 ± 2.82	-0.27 ± 2.14

### Profiles of three age-dependent subpopulations with differed health decline nationally (Step 3 shown in Fig. 1, upper)

Based on the scored health decline, older adults were further stratified into three subpopulations with the least health decline (score<-1.34), a moderate health decline (-1.34≤score≤-1.03) and the largest health decline (score>-1.03). These three subpopulations were further compared by age, gender, setting, residency, educational attainment and personal income (Table 4).

**Table 4:** Characteristics of subpopulations with different extent of ageing-dependent health burden in the older Chinese adults

Variables	All	Subpopulations with different health decline (Percentage distribution)				
		< -1.34 (least decline) *n* = 2,694	-1.34 to -1.03 (moderate decline) *n* = 2,702	> -1.03 (largest decline) *n* = 2,697	N	Statistics (*p* value)
Age					8,093	1008.05 (*p* < 0.001)
60–69	3,348 (41.37%)	1,597 (59.28%)	1,122 (41.52%)	629 (23.32%)		
70–79	3,185 (39.35%)	879 (32.63%)	1,177 (43.56%)	1,129 (41.86%)		
≥ 80	1,560 (19.28%)	218 (8.09%)	403 (14.91%)	939 (34.82%)		
Sex					8,093	3.53 (*p* = 0.17)
Female	3,786 (46.78%)	1,222 (45.36%)	1,274 (47.15%)	1,290 (47.83%)		
Male	4,307 (53.22%)	1,472 (54.64%)	1,428 (52.85%)	1,407 (52.17%)		
Residency					8,093	262.78 (*p* < 0.001)
Urban	6,265 (77.62%)	2,132 (79.37%)	2,102 (77.94%)	2,031 (75.56%)		
Rural	1,806 (22.38%)	554 (20.63%)	595 (22.06%)	657 (24.44%)		
Source/setting					8,071	11.50 (*p* = 0.003)
Community	3,885 (48.00%)	1,579 (58.61%)	1,319 (48.82%)	987 (36.60%)		
Hospital	4,208 (52.00%)	1,115 (41.39%)	1,383 (51.18%)	1,710 (63.40%)		
Educational attainment (Years)					7,924	228.61 (*p* < 0.001)
≤ 5	2,138 (26.98%)	467 (17.91%)	702 (26.50%)	969 (36.32%)		
6–9	1,596 (20.14%)	573 (21.98%)	539 (20.35%)	484 (18.14%)		
≥ 9	4,190 (52.88%)	1,567 (60.11%)	1,408 (53.15%)	1,215 (45.54%)		
Personal Income (Quartile)					6,872	112.58 (*p* < 0.001)
Lowest	1,721 (25.04%)	513 (19.80%)	597 (26.84%)	611 (29.70%)		
Second	1,712 (24.91%)	611 (23.58%)	592 (26.62%)	509 (24.74%)		
Third	1,712 (24.91%)	756 (29.18%)	553 (24.87%)	403 (19.59%)		
Highest	1,727 (25.13%)	711 (27.44%)	482 (21.67%)	534 (25.96%)		

### Validating two metrics (health decline and multimorbidity pattern) by Gradient distribution in the older adults (Arrow between Step 1 and Step 2 shown in Fig. 1, black block)

**Fig. 4:**
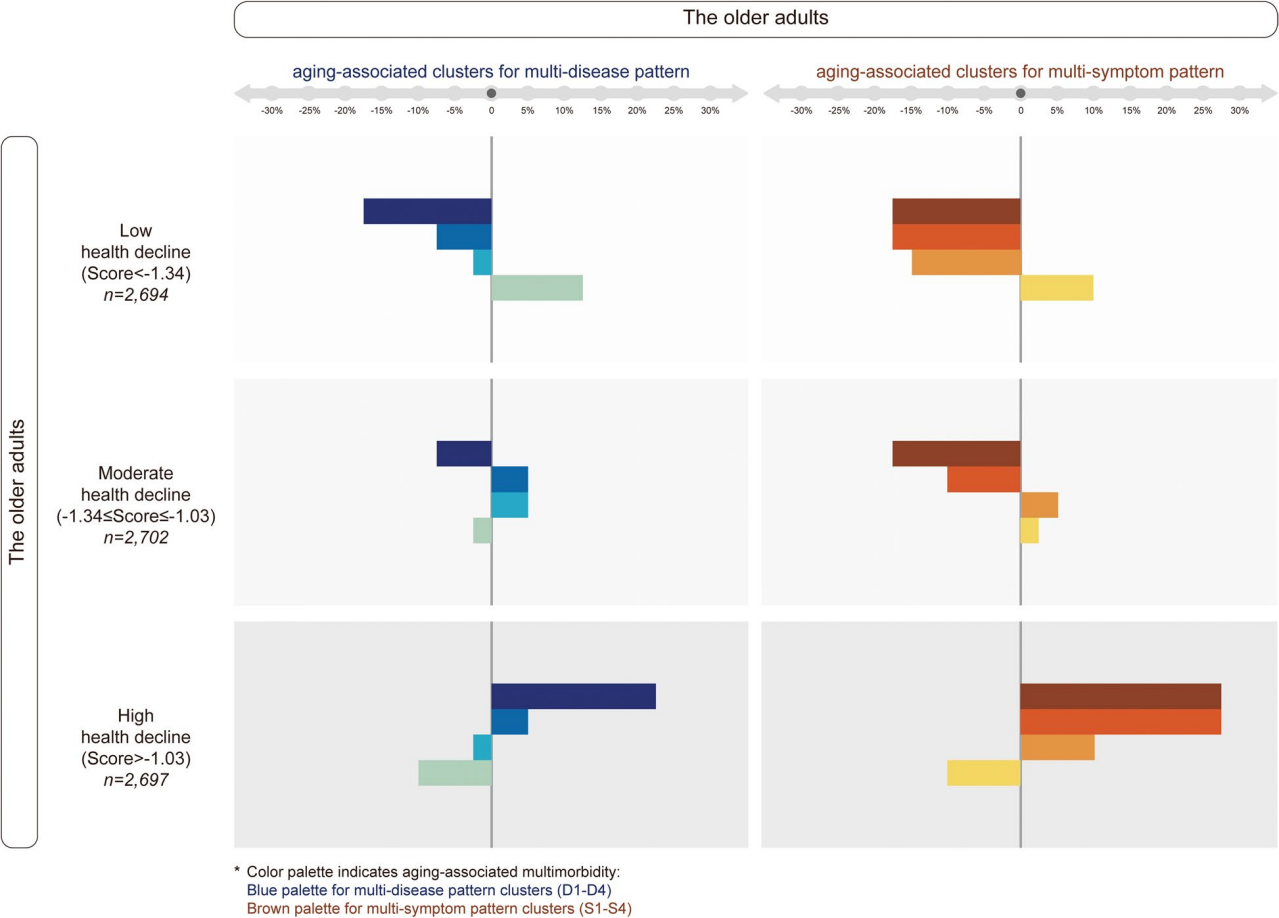
Distribution consistency of the subpopulations stratified between by health decline score and by multi-disease or multi-symptom pattern. Subpopulations harboring different age-dependent health decline and harboring different aging-associated multi-disease patten or multi-symptom patten were highly consistent, which was shown using a percentage-dependent gradient correlation. It meant aging-increased multimorbidity (depicted by color palette) was distributed in parallel to the increased health decline (depicted by grey palette), and vice versa

Percentage distribution was compared in three categorized subpopulations between by multimorbidity pattern cluster and by health decline score (Fig. 4). Considerable gradient consistency was shown in three subgroups with differed age-dependent health decline and in three subgroups with differed aging-associated multi-disease patten or multi-symptom patten. This outcome strongly validated these two metrics developed in the older adults.

### Profiles of the new datamined variable of difference between multi-disease score and multi-symptom score (DMM) nationally and sub-nationally

**Fig. 5:**
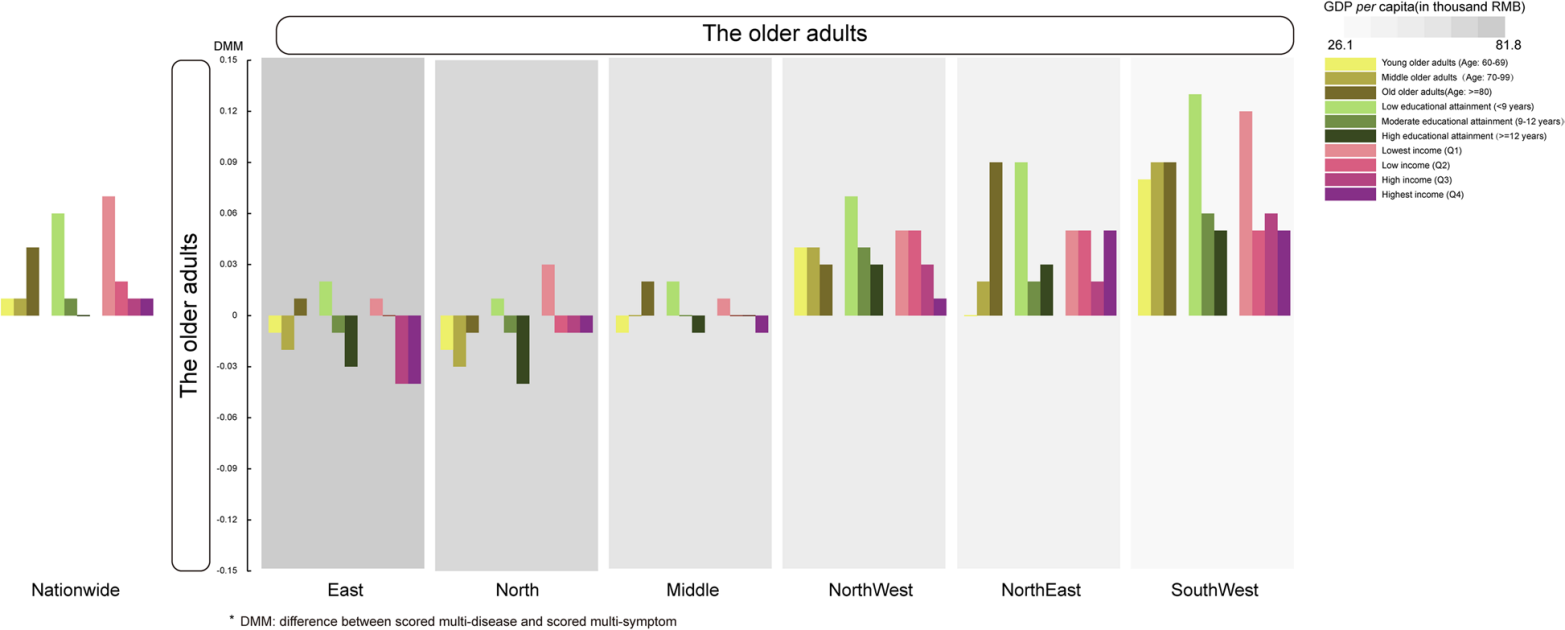
Variation of the difference between multi-disease score and multi-symptom score (DMM) by personal educational attainment and regional socioeconomic status. Decreased personal education was found linearly varied with increased DMM with minor exception. Also, more affluent regional socioeconomical status stratified by GDP per capital (depicting via grey palette) was found linearly varied with decreased DMM. Collectively, it indicated that DMM might be a socioeconomics-related indicator during multidimensional health decline in older Chinese population

We supposed the datamined variable of difference between multi-disease score and multi-symptom might play as a potential “link” to socioeconomics-dependent healthcare access. Therefore, we profiled DMM by socioeconomic variables individually and regionally. We witnessed a linear variation of DMM by personal education and by regional socioeconomic status (Fig. 5). It was intriguing that similar tendency had been shown in a previous WHO SAGE study conducted in developing countries [32]. Therefore, we sought to use optimized DMM as a potential socioeconomic indicator which might disentangle the multidimensional complexity of health decline from within.

### Independent U-shaped relation among health decline, multi-disease score and multi-symptom score at the optimally stratified DMM (Step 4 shown in Fig. 1)

**Fig. 6:**
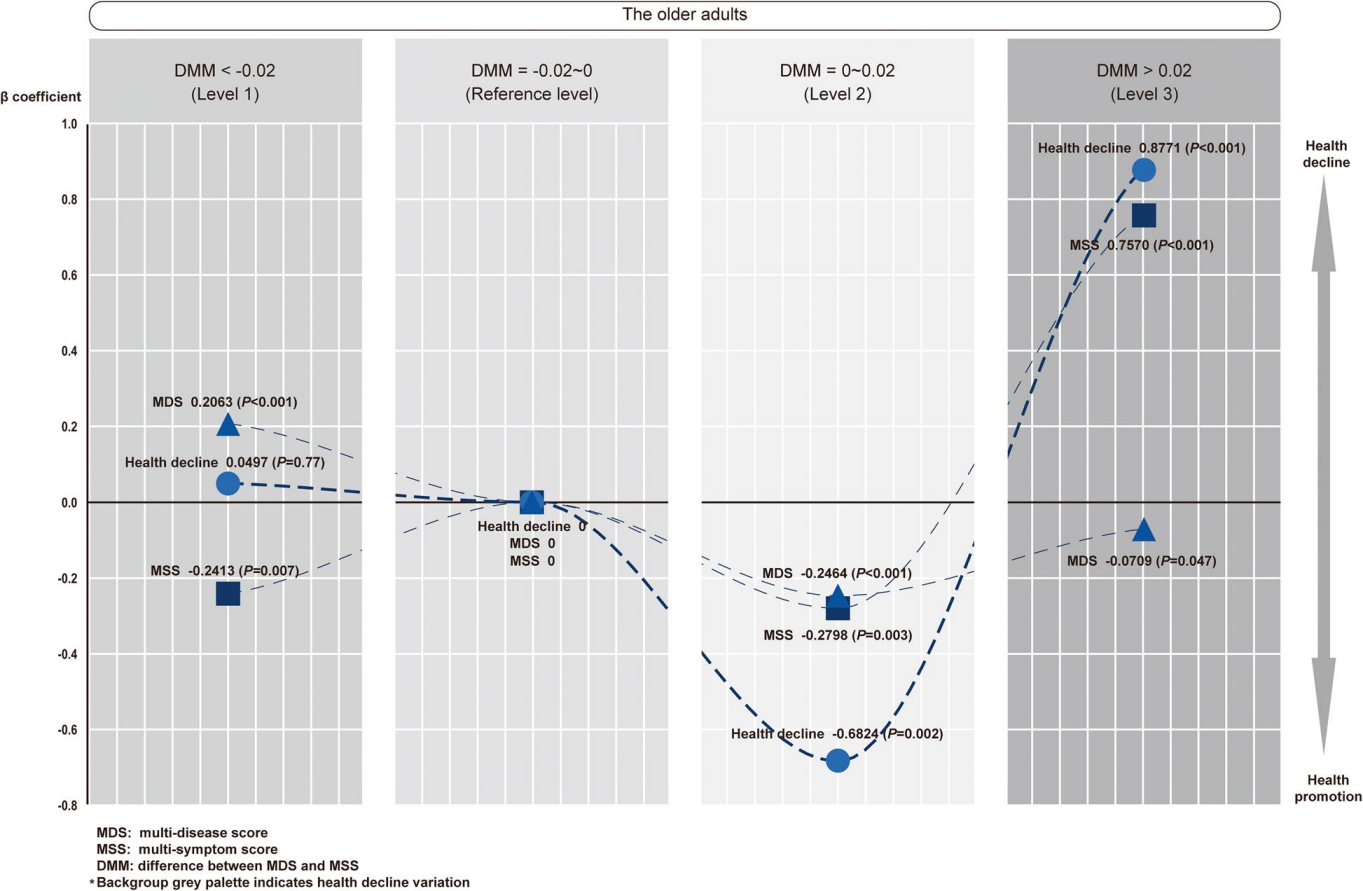
Independent U-shaped interrelated tendency between health decline score, multi-disease score or multi-symptom score at the optimally fitted DMM in the older adults by multilevel regression. A multilevel modelling was fitted with substantial adjustments by age, sex, setting, residency, education attainment, personal income (level-1) and region variation (level-2). After multiple fitting iterations, an optimization by various interval of DMM interval addressed DMM = 0.02 as optimal, based on which an intriguingly independent U-shaped interrelated tendency was shown between health decline score, multi-disease score and multi-symptom score. Compared to the reference subgroup (DMM at-0.02 ~ 0, health decline β = 0, MDS β = 0, MSS β = 0), the Level-1 subgroup demonstrated insignificantly yet mildly reduced health decline (β = 0.0497, *P* = 0.77) by a moderate increase in multi-diseases score (β = 0.2063) and a significant reduction in multi-symptom score (β = -0.2413). Significantly decreased health decline (β = -0.6824, *P* = 0.002) is shown in the subgroup Level-2 with multi-disease score and multi-symptom score in parallel; Level-3 subgroup addressed the significantly increased health decline (β = 0.8771, *P* < 0.001) with the highest multi-symptom score (β = 0.7570, *P* < 0.001) and unchanged multi-disease score comparing with the reference (*P* = 0.471)

As we revealed that the difference between MDS and MSS (DMM) is closely linked to socioeconomic variables, we used multilevel regressions to determine if an optimal DMM categorization aiding in disentangling the complicated interrelationship among health decline, multi-disease score and multi-symptom score. After multiple fitting iterations, an optimal stratification of DMM (DMM=0.02) was acquired, based on which an intriguingly independent U-shaped interrelated tendency between health decline, MDS and MSS was revealed (Fig. 6) by substantial adjustment of age, sex, setting, residency, education and income individually and variation regionally (Table 5). Importantly, such a U-shaped interrelated tendency could be well explained healthcare inequities based on the variation of subnational socioeconomic status in China. Regions with a higher MDS (indicating more disease awareness and better healthcare delivery) than MSS indicated the presence of superfluous healthcare, which occurred in affluent regions and is commonly seen in developed metropolitan areas in China [33]. However, it did not result in a lower health decline (*P*=0.77compared with the reference subgroup), indicating a low cost-effectiveness of health-care overutilization. In contrast, regions with a higher MSS than MDS reflected the presence of inadequate healthcare, which commonly occurred in relatively deprived regions, suggesting a markedly aggravated health decline (*P*<0.001). Similarly, an MDS similar to the MSS might indicate appreciated healthcare, which occurred in economically moderately developed regions (*P*=0.002), leading to cost-effective performance in healthcare delivery. Therefore, we considered the interval of DMM as optimal (DMM=0.02). Using DMM=0.02 as categorization, health decline score, multi-diseases score and multi-symptom score linearly increased with advanced age, particularly in the old-old subpopulation. Moreover, the most marked increase in health decline was seen in the hospital setting comparing with its counterpart, strongly validating the multilevel model.

**Table 5:** Interrelated tendency among health decline score, multi-disease score and multi-symptom score at an optimal DMM categorization using multilevel regression

Determinants	Levels	Health decline score		Multi-disease score		Multi-symptom score	
		β (SE)	*P*	β (SE)	*P*	β (SE)	*P*
DMM (Difference, MDS-MSS)	0:-0.02–0 (*Ref*)						
	1: < -0.02	0.0497(0.1702)	0.77	0.2063(0.0329)	< 0.001	-0.2413(0.0764)	0.007
	2: 0–0.02	-0.6824(0.1785)	0.002	-0.2464(0.0347)	< 0.001	-0.2798(0.0791)	0.003
	3: > = 0.02	0.8771(0.1694)	< 0.001	-0.0709(0.0327)	0.047	0.7570(0.0762)	< 0.001
Age	0: 60–69 (*Ref*)						
	1: 70–79	0.4163(0.2523)	0.13	0.0912(0.0346)	0.03	0.1392(0.0582)	0.04
	2: > = 80	1.4924(0.2590)	< 0.001	0.2328(0.0365)	< 0.001	0.4454(0.0618)	< 0.001
Sex	0:Female (*Ref*)						
	1: Male	-0.2301(0.0564)	0.010	-0.0275(0.0167)	0.16	-0.0683(0.0304)	0.07
Setting	0: Community (*Ref*)						
	1: Hospital	1.1771(0.1685)	< 0.001	0.1840(0.0549)	0.02	0.2779(0.1042)	0.04
Residency (Hukou)	0: City (Ref)						
	1: Rural	-0.0523(0.2452)	0.84	-0.0316(0.0178)	0.14	-0.0271(0.0287)	0.39
Educational attainment (Years)	0: < = 5 (*Ref*)						
	1: 6–9	-0.2424(0.0974)	0.03	-0.0092(0.0154)	0.56	-0.0502(0.0278)	0.10
	2: > = 10	-0.4035(0.0965)	0.002	0.0043(0.0154)	0.78	-0.0322(0.0279)	0.27
Personal Income (RMB Yuan)	0: < = 1800 (*Ref*)						
	1: 1800–3000	0.0679(0.1263)	0.60	0.0687(0.0316)	0.046	0.0952(0.0598)	0.13
	2: 3000–4000	-0.1938(0.1332)	0.17	0.0252(0.0327)	0.45	-0.0021(0.0618)	0.97
	3: > = 4000	-0.1518(0.1404)	0.30	0.0359(0.0339)	0.31	0.0298(0.0638)	0.65

## Discussion

In this study, we attempted to disentangle the complexity of age-dependent multi-dimensional health decline, particularly focusing on multimorbidity (multi-disease and multi-symptom) characterized as one of core constituted domains of health, in a population-based sample harboring 8,093 older Chinese adults from six subnational regions. For overall health decline, a widely used outcome of SRH, which exhibits an independent correlation with each of the health domains [34], was applied to adjust each score on respective domain within the multidimensional complexity of health decline [9]. Additionally, the component domains of age-dependent health decline have been documented to be interrelated [13], reciprocally entangled [1, 11, 35], contextually attenuated [20], and even paradoxically confounded by some socioeconomical variable [20]. To resolve this challenge, we standardized all SRH-adjusted scores before summation to avoid bias resulting from exclusive focus on any single domain of age-dependent health decline [34]. For multimorbidity coexistence, we acquired the SRH-adjusted MDS based on concomitant diagnosed chronic diseases [36] and the SRH-adjusted MSS based on concomitant perceived chronic symptoms. For multimorbidity patten, we used latent-based clustering to reduce the formidably heterogeneous multimorbidity pattern, and optimized the outcome cluster using BIC datamining. We further categorized outcome multi-disease pattern clusters or multi-symptom pattern clusters into three ageing-associated sub-groups. We next validated two newly developed metrics by gradient distribution in the old sample, and we saw a gradient consistency. For socioeconomical dimension of age-dependent health decline, we used two individual variables (educational attainment and personal income) and one regional variable (gross domestic product/capital) to correlate the overall health decline, multi-disease score and multi-symptom score. The profiles displayed an interesting inconsistency: high multi-disease score whereas low multi-symptom score correlating with educational attainment individually and with socioeconomical level regionally. The similar phenomenon had also been documented in other developing countries [32]. Therefore, we revealed an interesting new variable of the difference between multi-disease and multi-symptom, terming DMM. We found that DMM linearly varied to personal educational attainment as well as to regional socioeconomic level. Due to this phenomenon, we sought to “integrate” the multidimensional health decline from within with the aid of the new variable (DMM). We iteratively fitted DMM as varied intervals, until DMM=0.02 as the optimal via multilevel regression modelling. We revealed an independent U-shaped tendency among health decline score, multi-disease score and multi-symptom score at an optimal DMM=0.02 categorization after substantial adjustments. More interestingly, the U-shaped interrelated tendency could be well explained by diverse socioeconomic status between subnational regions: comparing to the reference group with medium health decline, multi-disease and multi-symptom (all score=0), a significantly reduced health decline appeared when the MDS was similar to the MSS, indicating appropriate healthcare delivery; a high MDS with a low MSS did not significantly decline health, which was likely an outcome of redundant healthcare with a low cost-effectiveness of healthcare delivery; a low MSS with a high MDS produced the largest health decline, largely caused by insufficient healthcare due to low socioeconomic level. It is extremely difficult to search an optimal scale to reduce healthcare delivery with respect to the comprehensive health decline with highly dynamic nature in older adults. In the present study, we revealed that an optimal fitted stratification of difference between multi-disease score and multi-symptom score, here termed DMM, could well explain the socioeconomic status-related healthcare delivery inequity nationally. Different from the traditional linear relationship, such stratification of DMM suggested a novel non-linear interrelationship, which was supposed to be consist to the real world. Within the multidimensional health decline during aging, the correlation between multi-diseases and multi-symptoms, both adjusted by self-rated health, were highly dependent to the personal and regional socioeconomic status [37, 38]. Therefore, an optimal interval of DMM=0.02 could help to disentangled the complexity of health decline and improve the cost-effectiveness in healthcare delivery in China nationally. For the East China which sampled from the top developed metropolitan of Shanghai in China, the excessive access of advanced healthcare did not achieve better health being but a slight health decline comparing to the reference based on Middle China (*P*>0.05). The complicated decline was explained by the notably increased multi-symptom score (*P*<0.05) which could not be well attenuated by mildly decreased multi-disease score (*P*<0.05). High social stresses, ongoing intense lifestyle and high expectancy on wellbeing might increase the mental burden and SRH-adjusted symptoms in the older adults in highly industrialized metropolitan, with overall health decline intact. For Northeast China with relatively lower socioeconomic status comparing to the reference region, the health decline was significantly mitigated, largely explained by both decreased multi-symptom score and decreased multi-disease score. It was noted that the minus magnitude of health decline was much higher than that summing minus magnitude of multi-disease and multi-symptom, indicating a high cost-effectiveness of healthcare access. For the Northwest China with much lower socioeconomic status comparing to the reference region, the health decline was significantly aggravated, largely explained by considerably high multi-symptom score, paradoxically with decreased multi-disease score. It indicates an attributable insufficiency of healthcare access lacking disease awareness, with high likelihood of no management. The outline of the U-shaped tendency indicated that the overall health decline was exponentially functioned with the multiple coexistence of multi-diseases and multi-symptoms. Due to the present analysis, the optimal interval of DMM could not only help to disentangle the complexity from within the contextual complexity of health decline, but offer an alternative scale for public health policies to reduce healthcare access inequity in the old adults. China is experiencing undergoing inequality in healthcare delivery and access [39, 40], demanding such indicator as DMM to improve the cost-effectiveness on public health distribution. It was noted that nation-based fitting was required for achieving the optimal categorization of DMM in other developing countries. To our best knowledge, the DMM is the first indicator that was developed on healthcare delivery inequity characterized with ①relative low cost and high efficiency in old adults;②indicator from within connecting individual-based clinical phenotypes which was supposed to partially secondary to industrialization-based healthcare delivery;③an innovative indicator which could be explained with respect to the intrinsic interrelation between three dimensions of health.

There are several weaknesses in the study. First, the analysis was conducted based on cross-sectional datasets with no cohort validation, as the complexity of multidimensional health decline with contextual variation highly hampered this. Second, the sample size was relatively small regarding to the largest ageing population in the world. Third, other ageing-related health burdens, such as disability, were not considered because very few disabled individuals were found in the original datasets. Fourth, the lifelong risk factors were not cumulated into the burden of multimorbidity coexistence, as it was an age-attenuated [41] and age-modified [42] associations of traditional risk factors with morbidity and mortality during aging. Fifth, to our best knowledge, no 3D health-based indicator on healthcare delivery was developed in aging population, we could not validate this innovative indicator externally. Only internal validation was performed.

## Conclusion

Two metrics were obtained for age-dependent health decline and for aging- associated multimorbidity pattern in the older adults in China, being validated from within. It was revealed a new variable of difference between multi-disease score and multi-symptom score characterized with a socioeconomic trait. An independent U-shaped tendency was shown between health decline, multi-disease and multi-symptom at an optimally fitted categorization of DMM in older Chinese adults, which was well explained by regional socioeconomic healthcare delivery inequities. The categorization of an optimal DMM might be a practical indicator to estimate whether an ageing-related healthcare inequality exist and how healthcare delivery be cost-effective in ageing developing countries.

### Supplementary Information

**Additional file 1: Supplementary File S1. **The compound scales designed for the National project for Comprehensive Ageing Health Assessment (CAHA) in 2012 (English Version) A compound scale comprising eight components was constructed based on Comprehensive Geriatrics Assessment (CGA) framework by a panel of multidisciplined experts for a national population-based survey. English version was particularly translated from the original Chinese version for the present publication. **Supplementary File S2.** The national sampling profile in Comprehensive Ageing Health Assessment (CAHA) in China, 2012 A goal-guided national project was carried out in six subnational regions covering approximately two-third of Chinese population and nearly 80% of China’s GDP in 2012 (Data from China National HYPERLINK "http://data.stats.gov.cn/"Database)http://data.stats.gov.cn.The central cities in these regions included three centrally-administrated municipalities (Beijing in North China; Shanghai in East China; Chongqing in Southwest China) and four capital cities (Harbin, Heilongjiang province in Northeast China; Chengdu, Sichuan province in Southwest China; Xi’an, Shanxi province in Northwest China; Changsha, Hunan province in Central China). A constructed scale was used for the national assessment with Department/Ward of 13 top hospitals. The distribution details of sampling numbers and settings are presented in Supplementary File S2. 

**Additional file 2.**

## Data Availability

Enlargeable PDF edition for each figure is available from corresponding author when required.

## Abbreviation

MDS: Multi-disease score
MSS: Multi-symptom score
SRH: Self-rated health
DMM: Difference between multi-disease score and multi-symptom score
LCA: Latent clustering analysis
BIC: Bayesian information criterion
CAHA: Comprehensive ageing health assessment
