# Supplementary material for: Could an optimally fitted categorization of difference between multi-disease score and multi-symptom score be a practical indicator aiding in improving the cost-effectiveness of healthcare delivery for older adults in developing countries?
Source: Int J Equity Health. 2023 Oct 11;22:213. doi: 10.1186/s12939-023-02024-z (PMC10568876; doi:10.1186/s12939-023-02024-z)
Supplement: Supplementary file 1 — Additional file 1: Supplementary File S1. The compound scales designed for the National project for Comprehensive Ageing Health Assessment (CAHA) in 2012 (English Version) A compound scale comprising eight components was constructed based on Comprehensive Geriatrics Assessment (CGA) framework by a panel of multidisciplined experts for a national population-based survey. English version was particularly translated from the original Chinese version for the present publication. Supplementary File S2. The national sampling profile in Comprehensive Ageing Health Assessment (CAHA) in China, 2012 A goal-guided national project was carried out in six subnational regions covering approximately two-third of Chinese population and nearly 80% of China’s GDP in 2012 (Data from China National HYPERLINK "http://data.stats.gov.cn/"Database)http://data.stats.gov.cn.The central cities in these regions included three centrally-administrated municipalities (Beijing in North China; Shanghai in East China; Chongqing in Southwest China) and four capital cities (Harbin, Heilongjiang province in Northeast China; Chengdu, Sichuan province in Southwest China; Xi’an, Shanxi province in Northwest China; Changsha, Hunan province in Central China). A constructed scale was used for the national assessment with Department/Ward of 13 top hospitals. The distribution details of sampling numbers and settings are presented in Supplementary File S2. [file 12939_2023_2024_MOESM1_ESM.pdf]

Serial Number□□□ □□□□□ NO

Name ______________________________Z1

Personal Number □□□□□□□□ Z2

Medical Card Number □□□□□□□□ Z3

Date of Examination □□□□/□□/□□

Y M D

Investigator Number □□ Z4

Reviewer Number □□ Z5

Comprehensive Assessment Scale of Geriatric Health

Ministry of Health Industry Fund Geriatric Health Comprehensive Qssessment Research Group

China Federation of Geriatric Health Care and Disease Control
2011

Detailed Address: ______Province_______City________Region_______Neighborhood

Committee_____________Street(Lane)_________Number

________County______Township______Village

Postal Code: □□□□□□

Personal Contact Information: Mobile Phone_____________TEL1 Home Phone____________TEL2

Name of Guardian (Contact): __________________Phone or Mobile: __________________

Identity Card Numbers □□□ □□□ □□□□ □□□□ □□□□ ID

Information Source: 1=Self 2=Family Member 3=Nurse 4=Others □ Z6

Subject Source: 1=Community 2=Medical Examination Center 3=Outpatient Department
4=Comprehensive Ward 5=Neurology Department 6=Cardiology Department
7=Other Medical System Ward 8=Other Surgical Ward 9=Other □ Z7

A Basic Information

Code

A01 Gender : 1= Male 2=Female ☐A1

A02 Birthdate: ______________________ ☐☐☐☐ ☐☐ ☐☐A2

Age: ________years old (Zodiac________) ☐☐☐A3

A03 Degree of Education: 1=illiteracy 2=Elementary School

3=Junior High School 4=Senior High School/Secondary Specialized School

5=Junior College、Bachelor 6=Master ☐A5

A04 N ationality: 1=Han 2=Mongolian 3=Hui 4=the Tibetan Ethnicity

5=the Korean Nationality 6=Others____________ ☐A6

A05 Marital Status: 1=Unmarried 2=Living together in marriage

3=Separationin marriage 4=Divorce

5=Bereft of one's spouse 6=Others ☐A7

A06 Former Occupation: 1=Worker 2=Peasant 3=Science and technology

4=Public functionary/Administrative cadre

5=Commercial service 6=Teacher 7=medical personnel

8=Housekeeping 9=Soldier 10=Individual business

11=Housework 12=Others (give clear indication of) ____ ☐☐A8

A07 What you have been working on in your life is:

1=Mental labor-oriented work

2=Light physical labor-oriented work

3=Heavy physical labor as the main work 4=Others ☐A9

A08 Family type:

1=Live alone 2=The couple live together

3=Two generations of households 4=Three generations of households

5=Intergenerational household 6=Others ☐A10

A09 Your average monthly income is_____yuan? (Fixed monthly, including children, etc.)

☐☐☐☐☐A11

A10 Residential address: 1=large and medium-sized cities 2=Suburb

3=County town 4=countryside

5=Others ☐A12

B Physical Health Assessment

B01 How do you feel about your health now? □B1

1=very good 2=good 3=moderate 4=bad 5=very bad

B02 The reason why you feel ill is that (Answer 4 or 5 to ask this question)

1=Have heavier chronic diseases.

2=No serious illness, but there are many uncomfortable symptoms.

3=No illness, just old age. □B2

4=Flexibility of legs and feet, inconvenience of movement.

5=I can't speak clearly.

B03 Existing chronic diseases (Diagnostic determination in medical institutions): 1=Yes 2=No □B3

	1=Yes 2=No	Years of illness	Treatment status			
Hypertension	□B4	□□B5	1=Untreated	2=intermittent	3=adherence	□B6
Coronary heart disease	□B7	□□B8	1=Untreated	2=intermittent	3=adherence	□B9
Other cardiovascular diseases	□B10	□□B11	1=Untreated	2=intermittent	3=adherence	□B12
Chronic obstructive pulmonary disease (COPD)	□B13	□□B14	1=Untreated	2=intermittent	3=adherence	□B15
Other respiratory diseases	□B16	□□B17	1=Untreated	2=intermittent	3=adherence	□B18
Gastrointestinal diseases	□B19	□□B20	1=Untreated	2=intermittent	3=adherence	□B21
Liver diseases	□B22	□□B23	1=Untreated	2=intermittent	3=adherence	□B24
Kidney diseases	□B25	□□B26	1=Untreated	2=intermittent	3=adherence	□B27
Stroke (thrombus、haemorrhage)	□B28	□□B29	1=Untreated	2=intermittent	3=adherence	□B30
Trantient ischemic attack (TIA)	□B31	□□B32	1=Untreated	2=intermittent	3=adherence	□B33
Dementia	□B34	□□B35	1=Untreated	2=intermittent	3=adherence	□B36
Other neurological diseases	□B37	□□B38	1=Untreated	2=intermittent	3=adherence	□B39
Psychiatric diseases	□B40	□□B41	1=Untreated	2=intermittent	3=adherence	□B42

Diabetes ☐B43 ☐☐B44 1=Untreated 2=intermittent 3=adherence ☐B45

Bone and joint diseases

☐B46 ☐☐B47 1=Untreated 2=intermittent 3=adherence ☐B48

Tumors ☐B49 ☐☐B50 1=Untreated 2=intermittent 3=adherence ☐B51

Cataract ☐B52 ☐☐B53 1=Untreated 2=intermittent 3=adherence ☐B54

Deafness ☐B55 ☐☐B56 1=Untreated 2=intermittent 3=adherence ☐B57

Others________ ☐B58 ☐☐B59 1=Untreated 2=intermittent 3=adherence ☐B60

B04 Is there a disability or disorder? 1=No 2=Yes ☐B61

B04.1 What kind of handicap is there?

Limb disability/defect 1=No 2=Yes ☐B62

Intelligence disability (moronism) 1=No 2=Yes ☐B63

Organ disability (important visceral resection/replacement) 1=No 2=Yes ☐B64

Psychic disability (psychic disorder for more than a year) 1=No 2=Yes ☐B65

Vision disorder (corrected visual acuity less than 0.3) 1=No 2=Yes ☐B66

Dysaudia (severe hearing impairment in both ears) 1=No 2=Yes ☐B67

Speech disorder (aphasia) 1=No 2=Yes ☐B68

B05 Do your relatives have the following diseases?(Parents, brothers and sisters, children)

1 Dementia	1=No	2=Yes	☐B69
2 Coronary heart disease	1=No	2=Yes	☐B70
3 Hypertension	1=No	2=Yes	☐B71
4 Diabetes	1=No	2=Yes	☐B72
5 Stroke	1=No	2=Yes	☐B73
6 Psychiatric diseases	1=No	2=Yes	☐B74
7 Tumors	1=No	2=Yes	☐B75

B06 Do you have the following symptoms:

	1=No 2=Yes	How many years	Whether to treat		
Chest tightness or chest pain	☐B76	☐☐B77	1=No	2=Yes	☐B78
Dizziness	☐B79	☐☐B80	1=No	2=Yes	☐B81
Significant memory loss	☐B82	☐☐B83	1=No	2=Yes	☐B84

Sequela of apoplexy	☐B85	☐ ☐B86	1=No	2=Yes	☐B87
Transient slurred speech/Aphasia	☐B88	☐ ☐B89	1=No	2=Yes	☐B90
Recurrent joint pain	☐B91	☐ ☐B92	1=No	2=Yes	☐B93
Chronic cough/ wheezing	☐B94	☐ ☐B95	1=No	2=Yes	☐B96
Insomnia	☐B97	☐ ☐B98	1=No	2=Yes	☐B99
Fall more than twice recently a year	☐B100	☐ ☐B101	1=No	2=Yes	☐B102
Urinary incontinence	☐B103	☐ ☐B104	1=No	2=Yes	☐B105
Dysuria	☐B106	☐ ☐B107	1=No	2=Yes	☐B108
Constipation	☐B109	☐ ☐B110	1=No	2=Yes	☐B111
Spontaneous fracture after 50 years old	☐B112	☐ ☐B113	1=No	2=Yes	☐B114
Inactivity (slow/cripple)	☐B115	☐ ☐B116	1=No	2=Yes	☐B117
Exertional dyspnea /edema	☐B118	☐ ☐B119	1=No	2=Yes	☐B120

C Physical Function Assessment

C01 Can you do the following independently now?

Content	Degree of self-care<br>1 Complete self-care<br>2 Partial dependence<br>3 Absolute dependence	How many years can't take care of oneself		
Eat	_____	_____	☐C1	☐☐C2
Wash and comb your hair	_____	_____	☐C3	☐☐C4
Dress and undress	_____	_____	☐C5	☐☐C6
Up and down the bed	_____	_____	☐C7	☐☐C8
Take a shower	_____	_____	☐C9	☐☐C10
Indoor activity	_____	_____	☐C11	☐☐C12
Use the toilet	_____	_____	☐C13	☐☐C14
Cook	_____	_____	☐C15	☐☐C16
Management of property	_____	_____	☐C17	☐☐C18
Ride by yourself	_____	_____	☐C19	☐☐C20
Shopping on the street	_____	_____	☐C21	☐☐C22
Walk half a kilometer	_____	_____	☐C23	☐☐C24
Cut toenails	_____	_____	☐C25	☐☐C26
Up and down one floor	_____	_____	☐C27	☐☐C28

C02 Physical examination

C02.1 Stand with eyes open and feet together for 10 seconds, accomplish _________seconds ☐☐C29

C02.2 Stand with eyes closed and feet together for 10 seconds, accomplish______________seconds ☐☐C30

C02.3 Stand with eyes open and feet back and forth for 10 seconds, accomplish______________seconds ☐☐C31

C02.4 Stand up from your chair five times as quickly as possible

1=Completion 2=Partial completion 3=not completed ☐C32

C02.5 Daily walking speed turns back to 20 meters and time

1=Completion 2=Partial completion 3=not completed ☐C33

Completion time_________seconds ☐C33b

D Life Behavior and Social Function Assessment

D01 Do you have the habit of smoking?

1=No（Jump question D01.5） 2=Yes，frequent smoking

3=In the past, I have quit smoking (for more than half a year) ☐D1

D01.1 How many cigarettes do you smoke every day on average?_______cigarettes ☐☐D2

D01.2 How old are you to start smoking?_______years old ☐☐D3

D01.3 How many years have you smoked?_______years ☐☐D4

D01.4 If you have quit smoking, how old are you to quit smoking?_______years old ☐☐D5

D01.5 Do you smoke secondhand smoke regularly?(One day a week up to 15 minutes)

1=Yes 2=No ☐D6

D01.6 If so, the approximate age for your secondhand smoke is_______ years? ☐☐D7

D02 Do you drink?

1=No (Jump question D03) 2=Drink regularly

3=Drinking in the past, but now quit ☐D8

D02.1 How many years have you been drinking?_______ years ☐☐D9

D02.2 The most common drinks you drink are (choose only the main one）?

1=Liquor 2=Beer 3=Red wine 4=Others（ ） ☐D10

D02.3 Alcohol consumption： Liquor_______liang/time ☐☐D11

Wine _______ liang/time ☐☐D12

Beer_______ml/time(1 standard bottle=600ml, 1 zip-top can=350ml)

☐☐☐☐D13

D03 Do you often take part in physical exercise (outdoor activity) ?

1=More than 4 hours/day   2=2-3 hours/day (10-20 hours/week)

3=1 hour/day (6-10 hours/week)   4=Half an hour/day (3-5 hours/week)

5=Almost no participation   □D14

D04 Eating habits:
1 Salty 2 Sweet 3 Fried food 4Vegetarian diet 5 Spicy 6 No preference
1=Yes (Multiple selection)

□D15 □D15b □D15c □D15d □D15e □D15f

D05	Meat eating habit:	1=Often (almost every day)	2=More frequent (2-3 times per week)	3=Less	4=Vegetarian diet	□D16
D06	Egg eating habit:	1=Often (almost every day)	2=More frequent (2-3 times per week)	3=Less	4=Not to eat	□D17
D07	Fish eating habit:	1=Often (almost every day)	2=More frequent (2-3 times per week)	3=Less	4=Not to eat	□D18

D08 Drinking habit: 1=No hobby 2=Drink coffee regularly

3=Drink a lot of drinks 4=Drink tea regularly □D19

D09 Sleep habit: 1=Less than 6 hours a day 2=6-8 hours a day

3=More than 8 hours a day □D20

D10 Sleep quality (self-perception): 1=Good 2=Commonly 3=Poor □D21

D11 Regular life: 1=Very regular 2=Basic regular 3=Irregular □D22

D12 Do you help with housework (including taking care of children) ?

1=Often 2=Sometimes 3=Not to do □D23

D13 Working hours (including self-employed) that are still in employment:

1=Full-time work 2=Half-day or part-time work 3=Not working □D24

D14 Are you involved in community service, helping neighborhoods and other social welfare activities (including paid or unpaid)?

1=Regular participation 2=Sometimes participation

3=Non-participation □D25

E Mental health

E 01 Geriatric Depression Scale(GDS) (Feelings of the past week)

instruction: The following mental health test, please answer your feelings, no right or wrong.

1、 Are you basically satisfied with life? Yes=0 No=1 □E1

2、 Have you given up many activities and interests? Yes=1 No=0 □E2

3、 Do you feel that life is empty? Yes=1 No=0 □E3

4、 Do you often get tired of it? Yes=1 No=0 □E4

5、 Do you think there is hope for the future? Yes=0 No=1 □E5

6、 Are you upset because you can't get rid of some ideas in your mind? Yes=1 No=0 □E6

7、 Are you energetic most of the time? Yes=0 No=1 □E7

8、 Are you afraid that something bad will happen to you? □E8 Yes=1 No=0

9、 Do you feel happy most of the time? Yes=0 No=1 □E9

10、 Do you often feel isolated? Yes=1 No=0 □E10

11、 Do you often get restless and upset? Yes=1 No=0 □E11

12、 Do you want to stay at home rather than do something new? □E12 Yes=1 No=0

13、 Do you often worry about the future? Yes=1 No=0 □E13

14、 Do you think your memory is worse than before? Yes=1 No=0 □E14

15、 Do you think you are enjoying your life now? Yes=0 No=1 □E15

16、 Do you often feel heavy and depressed? Yes=1 No=0 □E16

17、 Do you think it's meaningless to live like this? Yes=1 No=0 □E17

18、 Do you always worry about the past? Yes=1 No=0 □E18

19、 Do you find life exciting? Yes=0 No=1 □E19

20、 Is it difficult for you to start a new job? Yes=1 No=0 □E20

21、 Do you think life is full of vitality? Yes=0 No=1 □E21

22、 Do you think your situation is hopeless? Yes=1 No=0 □E22

23、 Do you think most people are much better than you? Yes=1 No=0 □E23

24、 Do you often grieve over trifles? Yes=1 No=0 □E24

25、 Do you often feel like crying? Yes=1 No=0 □E25

26、 Do you have any difficulty concentrating? Yes=1 No=0 □E26

27、 Are you happy to get up in the morning? Yes=0 No=1 □E27

28、 Do you want to avoid the party? Yes=1 No=0 ☐E28

29、 Is it easy for you to make a decision? Yes=0 No=1 ☐E29

30、 Is your mind as clear as usual? Yes=0 No=1 ☐E30

E02 Do you have the following information?

Think of the good in the face of bad things.	1= Yes	2= No	☐E31
It's easy to get nervous and worried about trifles.	1= Yes	2= No	☐E32
Like to make friends.	1= Yes	2= No	☐E33
Memory is much worse than before.	1= Yes	2= No	☐E34
Every change of environment, often not used to, it is difficult to adapt.	1= Yes	2= No	☐E35

E03 You feel about your current life:

1=Very interesting 2=It's kind of interesting 3=Commonly

4=No fun(boring) 5=It's boring (it's boring to live) ☐E36

E04 Are you satisfied with your health?

1=Very satisfied 2=Satisfied 3=Commonly

4=Dissatisfied 5=Very dissatisfied ☐E37

E05 Are you satisfied with your life?

1=Very satisfied 2=Satisfied 3=Commonly

4=Dissatisfied 5=Very dissatisfied ☐E38

F Cognitive Function

F01 Mini-Mental State Examination（MMSE）

Question and Guidance	Grade	Completion	Score
1 Execute the Continuous Order: I'll give you a piece of paper. Please do as I say. "Pick up the paper with your right hand, fold it in half, and put it on your lap."	3	_____	□F1
2 Reading comprehension:Please read this sentence and do what it means. (Show a piece of paper with "Close your eyes").	1	_____	□F2
3 Name: (Show a watch) What's this? (Show a pen) What's this?	2	_____	□F3
4 Composition ability:(Show a pattern, Same original picture)Please draw like this.	1	_____	□F4

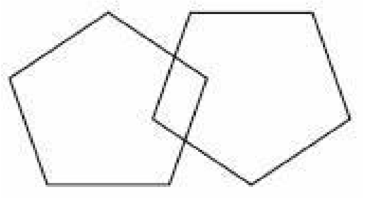

Close your eyes

Question and Guidance	Grade	Completion	Score
5 Write:Please write down your name.	1	_____	□F5
6 Memorizing:I'll tell you three things. Listen carefully, "Keys, cups, rulers." Please retell. Okay,please remember, I'll ask you later, please say it again.	3	_____	□F6
7 Temporal orientation : What day is today? What date? What month? What year? What season?	5	_____	□F7
8 Place orientation : Where are we now? What street is it on? How many floors is this (door number)? Which city? What country?	5	_____	□F8
9 Remember: Would you please recall the three things I just let you remember?	3	_____	□F9
10 Attention and calculation:Would you please calculate the number of 100-7? Then connect down to minus seven, and the number from you is reduced downwards (a total of five times)	5	_____	□F10
11 Attention and concentration:Please count down from 10 to 1.	1	_____	□F11

Total scores _____☐☐F12

F02 The degree of cooperation of the elderly in MMSE examination

1=Cooperate 2=Not very cooperative

3=Completely uncooperative ☐F13

G Medical Situation

G1 Have you been sick or unwell to see a doctor in the last two weeks?

1=Not sick 2=See a doctor if you are sick or unwell

3=Sick or uncomfortable but not see a doctor □G1

G1.1 Why don't you go and see it? (answer 3 to top question)

1=Financial difficulty

2=Too much trouble to see a doctor, too long to wait.

3=I can't go by myself. No one else has time to accompany me.

4=It can't see it well, I just quit going.

5=Take your own medicine to deal with it.

6=Others_____________________________ □G1A

G2 How often do you see a doctor in the past year (excluding hospitalization, including taking medicine)?

1=Once a week 2=Once every two weeks 3=Once a month

4=Once every two months 5=Once every three months

6=Occasionally or not □G2

G3 Have you ever been hospitalized in the last year?

1=Yes 2=No(jump question G4) □G3a

How many times have you lived in the hospital? _____ times □□G3

Total hospitalization days___________ □□□G4

G4 Did you stay in bed because of illness or discomfort for the last two weeks? 1=Yes 2=No □G5

G5 In the past year, have you needed the help of others because of your illness? 1=Yes 2=No(jump question G7) □G6

G6 Who mainly helps you (more than 50% of the situation)?

1=Spouse 2=Children 3=Grandchildren 4=Neighbors, friends (to help)
5=Nurse 6=Community Service Personnel

7=Senior Apartment or Home Service Personnel

8=Other Service Personnel (Charged) 9=Others_________ □G7

G7 Do you need to take medicine often because of chronic diseases? how many kinds do you take? ___________kinds □□G8

G8 Types of Medical Expense Payment:

1=Socialized medicine 2=Social medical insurance

3=Commercial Medical Insurance

4=New rural cooperative medical system

5=At one's own expense 6=Others □G9

G9 Proportion of reimbursement for your medical expenses______% □□□G10

G10 How much medical expenses have you spent in the last year?___yuan □□□□□G11

G11 Have you had a health check-up in the past year?

1=Yes 2=No □G1

H Evaluation scale of disability grade for the elderly

Please circle the corresponding grades.○

Title number	Item	Complete self-care	Require equipment assistance	Need prompt or help to prepare	Need somebody to give you a light hand(1/4)	Need moderate help from others(2/4)	Need someone else to give you a big hand(3/4)	Basically dependent on others	
1	Take food	7	6	5	4	3	2	1	☐H1
2	Wash up	7	6	5	4	3	2	1	☐H2
3	Go to the toilet	7	6	5	4	3	2	1	☐H3
4	Have a bath	7	6	5	4	3	2	1	☐H4
5	Put on/take off your coat	7	6	5	4	3	2	1	☐H5
6	Wear/take off your trousers	7	6	5	4	3	2	1	☐H6
7	Dispel stool	7	6	5	4	3	2	1	☐H7
8	Fecal incontinence and frequency	7	6	5	4	3	2	1	☐H8
9	Dispel urine	7	6	5	4	3	2	1	☐H9
10	Urinary incontinence and frequency	7	6	5	4	3	2	1	☐H10
11	Move between bed and chair/ wheelchair	7	6	5	4	3	2	1	☐H11
12	Get in and out of the toilet	7	6	5	4	3	2	1	☐H12
13	Get in and out of the bathroom/ tub	7	6	5	4	3	2	1	☐H13
14	Walk (or use a wheelchair)	7	6	5	4	3	2	1	☐H14
15	Up and down one stair	7	6	5	4	3	2	1	☐H15
16	Use of vehicles	7	6	5	4	3	2	1	☐H16
17	Shopping	7	6	5	4	3	2	1	☐H17
18	Do housework	7	6	5	4	3	2	1	☐H18
19	Personal property management	7	6	5	4	3	2	1	☐H19
20	Take medicine according to doctor's advice	7	6	5	4	3	2	1	☐H20
21	Problem solving ability	7	6	5	4	3	2	1	☐H21

22	Memory	7	6	5	4	3	2	1	☐H22
23	Directional ability	7	6	5	4	3	2	1	☐H23
24	Attention	7	6	5	4	3	2	1	☐H24
25	Emotional state	7	6	5	4	3	2	1	☐H25
26	Sociability	7	6	5	4	3	2	1	☐H26
27	Understanding ability	7	6	5	4	3	2	1	☐H27
28	Expressive ability	7	6	5	4	3	2	1	☐H28
29	Reasonable use of leisure time	7	6	5	4	3	2	1	☐H29
30	To be safely alone in a community, family or hospital	7	6	5	4	3	2	1	☐H30

L Auxiliary Examination

(Laboratory indicators in the past year)

L1 Weight______________kg □□□.□L1

L2 Height______________cm □□□L2

L3 Waistline___________cm □□□L3

L4 Hipline_____________cm □□□L4

L5 Blood pressure _____/______mm Hg □□□/□□□L5- L6

Is it normal:

1=Normal 2=Increase 3=Decrease

L6 Blood glucose GLU__________mmol/L □□.□□L7 □L7b

L7 2-hour postprandial blood sugar PBG_______mmol/L □□.□□L8 □L8b

L8 Glycosylated hemoglobin HbA1c___% □□.□L9 □L9b

L9 Triglyceride TG _________mmol/L □□.□L10 □L10b

L10 Total cholesterol TCH__________mmol/L □□.□L11 □L11b

L11 High density lipoprotein HDL____mmol/L □.□□L12 □L12b

L12 Low density lipoprotein LDL____mmol/L □.□□L13 □L13b

L13 Uric acid UA___________umol/L □□□L14 □L14b

L14 Creatinine CRE__________umol/L □□□.□L15 □L15b

L15 Urea nitrogen BUN__________mmol/L □□.□L16 □L16b

L16 Alanine aminotransferase ALT________Iu/L □□□L17 □L17b

L17 Aspartate aminotransferase AST_______Iu/L □□□L18 □L18b

L18 Creatine phosphate creatine enzyme CPK_______Iu/L □□□L19 □L19b

L19 Prealbumin PA___________mg/L □□□L20 □L20b

L20 Routine blood test:white blood cell WBC___mmol/m^3 □□□□.□L21

Red blood cell RBC_______mmol/m^3 □□□□.□L22

Platelet PT________mmol/m^3 □□□□.□L23

Hemoglobin Hb________mmol/L □□□□.□L24

L21 Electrocardiogram: Arrhythmia 1=Yes 2= No □L25

ST-T change 1=Yes 2= No □L26

Ventricular hypertrophy 1=Yes 2= No □L27

Old myocardial infarction 1=Yes 2= No □L28

L22 Echocardiography:

Left atrial diameter LA _________mm □□.□L29

Interventricular septal thickness ______mm □□.□L30

Left ventricular posterior wall thickness___mm □□.□L31

Left ventricular diameter LV________mm □□.□L32

Ejection fraction EF_________% □□.□L33

E/A ratio 1=<1.0 2=≥1.0 □L33b

Segmental wall dyskinesia 1=Yes 2=No □L34

L23 Bilateral carotid ultrasonography:

Right intimal-medial thickness_____mm □.□L35

Left intimal-medial thickness_____mm □.□L36

Plaque 1=Yes 2=No □L37

Plaque character:		
	Right	Left
Quantity	1=solitary 2=multiple □L38	1=solitary 2=multiple □L39
Homogeneity	1=Yes 2=No □L40	1=Yes 2=No □L41
Echo intensity	1=Low echo 2=Equal echo<br>3=Dense echo 4=Mixed echo<br>□L42	1=Low echo 2=Equal echo<br>3=Dense echo 4=Mixed echo<br>□L43
Initial plaque of internal carotid artery	1=Yes 2=No □L44	1=Yes 2=No □L45

Carotid stenosis (refers to ≥50% stenosis):

1=Right unilateral 2=Left unilateral 3=Bilateral 4=No □L46

Initial stenosis of internal carotid artery:

1=Yes 2=No □L47

Optional items (non-essential items):

L24 Lung function: Vital capacity VC ____ml □□□□.□L48

Time vital capacity FVC_______ml □□□□.□L49

Forced vital capacity in the first second

FEV1________ml □□□□.□L50

L25 Bone density: Hip: 1=Measure 2=Unmeasured □L51

BMD ____________g/cm^2 □□.□□L52

T-score _________ □□.□□L53

Z-score ________ □□.□□L54

Spine: 1=Measure 2=Unmeasured □L55

BMD ____________g/cm^2 □.□□□L56

T-score _________ □□.□□L57

Z-score ________ □□.□□L58

L26 Funduscope: Fundus arteriosclerosis 1=Yes 2=No □L59

Arterial morphology________

L27 Abdominal ultrasonography:

Fatty liver 1=Yes 2=No □L60

Hepatic cyst 1=Yes 2=No □L61

Renal cyst 1=Yes 2=No □L62

Others___________ □L63

L28 Chest radiograph emphysema 1=Yes 2=No □L64

L29 Prostate ultrasound hyperplasia of prostate gland 1=Yes 2=No □L65
