# Supplementary material for: Could an optimally fitted categorization of difference between multi-disease score and multi-symptom score be a practical indicator aiding in improving the cost-effectiveness of healthcare delivery for older adults in developing countries?
Source: Int J Equity Health. 2023 Oct 11;22:213. doi: 10.1186/s12939-023-02024-z (PMC10568876; doi:10.1186/s12939-023-02024-z)

Supplementary File S2. Sampling protocol and geographic distribution of 9,400 older Chinese adults from six subnational regions for Comprehensive Aging Health Assessment in China A goal-guided national project was carried out in six subnational regions covering approximately two-third of Chinese population and nearly 80% of China's GDP in 2012 (China National Database). The central cities in these regions included three centrally-administrated municipalities (Beijing in North China; Shanghai in East China; Chongqing in Southwest China) and four capital cities (Harbin, Heilongjiang province in Northeast China; Chengdu, Sichuan province in Southwest China; Xi'an, Shanxi province in Northwest China; Changsha, Hunan province in Central China). A total of 13 assessment centers were based on the Geriatrics Department/Ward of 13 top-leveled hospitals. The sampling numbers and settings are presented in Figure S1.

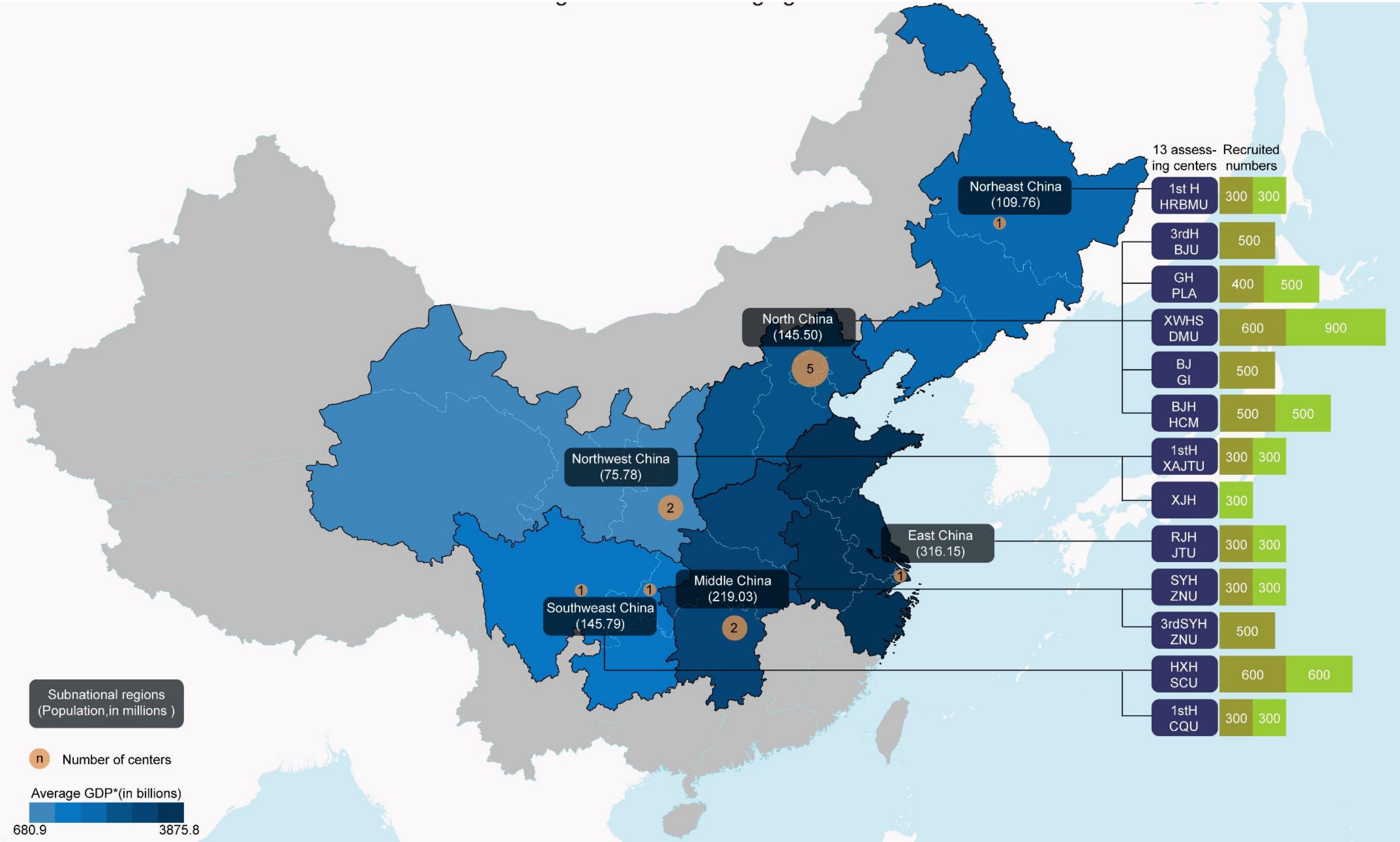

Supplement: Supplementary file 2 — Additional file 2. [file 12939_2023_2024_MOESM2_ESM.pdf]
